# P218: Active surveillance for central line associated bloodstream infections in hospitalized children in Greece

**DOI:** 10.1186/2047-2994-2-S1-P218

**Published:** 2013-06-20

**Authors:** K Mougkou, S Kouni, G Kurlaba, M Kitra, D Gkentzi, S Maroudi-Manta, A Lourida, S Coffin, T Zaoutis

**Affiliations:** 1 Athens School of Medicine, Collaborative Center for Clinical Epidemiology and Outcomes Research (CLEO), Greece; 2 Aglaia Kyriakou Children`s Hospital, Athens, Greece; 3 University General Hospital of Patras, Patra, Greece; 4 University of Pennsylvania School of Medicine , Division of Infectious Diseases The Children's Hospital of Philadelphia, Philadelphia, USA

## Introduction

Central line associated bloodstream infection (CLABSI) is the most common healthcare-associated infection in high-risk children and is associated with significant mortality, increased length of hospital stay and increased healthcare costs. There are limited data on the epidemiology of CLABSIs in hospitalized children in Greece.

## Objectives

The study aim was to prospectively assess the epidemiology of CLABSIs in 3 children`s hospitals in Greece.

## Methods

We conducted active surveillance for CLABSIs in 2 pediatric intensive care units (PICU), 5 neonatal intensive care units (NICU), 2 oncology units and the Bone Marrow Transplant Unit (BMTU) from September 2012 to February 2013. Surveillance was conducted using the Centers for Disease Control and Prevention definitions.

## Results

There were 34 CLABSIs (25 patients) within the study period resulting in an overall rate of 4.4/1.000 catheter-days. The highest rate was observed in PICUs (14.4) followed by NICUs (2.4), BMTU (1.9) and oncology units (1.8). The most common isolate was *Enterobacter* (20%), followed by *Klebsiella* (16%), *E.coli* and *Candida* (14%) and *Pseudomonas* (11%). Multi-drug resistant organisms predominated with 2/3 (66%) of *enterococci* exhibiting resistance to vancomycin and 15/24 (62%) of the Gram negatives were resistant to third generation cephalosporins (likely ESBLs). Carbapenem resistance was seen in 1/24 (4%) of Gram negative isolates.

Almost all patients had received antibiotics in the previous month (92%). 52% of the children who experienced a CLABSI were oncology patients. 56% of children with CLABSI had received parenteral nutrition and 76% blood transfusion within 7 days from the infection.

## Conclusion

We found high CLABSI rates in the PICU compared to rates reported in the United States and European Union. Rates in the other units were comparable to rates reported in the USA and EU. The majority of pathogens were MDROs and this is consistent with the high rate of MDROs, especially in adults, reported from Greece. Infection control and antimicrobial stewardship efforts should be developed and focused on units with high CLABSI and MDRO rates.

## Disclosure of interest

None declared

